# Congenital idiopathic talipes equinovarus before and after walking age: observations and strategy of treatment from a series of 88 cases

**DOI:** 10.1007/s10195-015-0377-4

**Published:** 2015-09-26

**Authors:** Cesare Faldini, Francesco Traina, Matteo Nanni, Ilaria Sanzarello, Raffaele Borghi, Fabrizio Perna

**Affiliations:** University of Bologna, Bologna, Italy; Dipartimento Rizzoli-Sicilia, Istituto Ortopedico Rizzoli, Strada Statale 113 km 246, 90011 Bagheria, PA Italy; University of Messina, Messina, Italy

**Keywords:** Clubfoot, Casting, Re-casting, Surgical treatment, Walking age

## Abstract

**Background:**

We reviewed a series of newborns, toddlers and ambulating children affected by idiopathic congenital talipes equinovarus (clubfoot). Taking into account the time of diagnosis, stiffness of the deformity and walking age, nonsurgical or surgical treatment was considered. This study reports clinical outcomes, early complications and relapse at mid-term follow-up.

**Materials and methods:**

Fifty-two clubfeet were diagnosed at birth, 12 in non-ambulating children aged between 4 and 12 months and 24 in ambulating children. Feet were classified using the Pirani score. Newborns and toddlers were treated with serial casting (Ponseti); however, toddlers also underwent open Achilles tendon lengthening (2 feet) and posteromedial release (3 feet). In all ambulating children, surgical treatment was always performed: selective medial release combined with cuboid subtraction osteotomy (1 foot), posteromedial release (6 feet), and posteromedial release combined with cuboid subtraction osteotomy (17 feet).

**Results:**

The average follow-up was 5 years (1–6 years). In newborns treated with Ponseti, the results were excellent in 42 feet, good in 6, and poor in 4. In non-ambulating children, the results were excellent in 9 feet, and good in 3. In ambulating children, the results were excellent in 5 feet, good in 16, and poor in 3. No major complications were reported. No overcorrections were observed. The need for open surgery was higher in cases of delayed treatment. In cases of relapse, re-casting and/or more extensive surgery was considered.

**Conclusions:**

Early treatment enables a high rate of good correction to be obtained with serial casting and limited surgery. Conversely, if the deformity is observed after walking age surgery should be considered. Serial casting in cases of late observation and relapse have demonstrated encouraging results.

**Level of evidence:**

IV.

## Introduction

Congenital idiopathic talipes equinovarus, also known as clubfoot, is a deformity present at birth that is characterized by a permanent alteration of the morphology of the foot and its relationship with the leg so the foot cannot lean on the ground in a physiological way. Therefore, the treatment should aim to correct all the components of the deformity (cavus, forefoot varus, hindfoot varus, equinus), in order to restore as much as possible of the physiologic morphology and function of the foot to allow plantigrade stance and proper gait.

If diagnosed at birth, clubfoot can be successfully treated nonsurgically. When performed on reducible deformities in newborns or toddlers, manipulations and serial casting, as described by Ponseti et al. [1], or manipulations and functional taping, as described by Masse et al. [2], can achieve successful correction in a high percentage of cases. In most cases, percutaneous Achilles tendon section often completes this kind of treatment in order to obtain correction of the equinus deformity. Over recent years nonsurgical treatment has been popularized due to excellent results reported by several authors [3–6].

However, if the deformity is diagnosed later or observed after unsuccessful conservative treatment, correction may require surgical treatment because of soft-tissue retraction, joint stiffness and bony changes that make the deformity more rigid as the child ages. A neglected or uncorrected deformity forces the child to start walking with the foot leaning on its lateral aspect [7]. The weight bearing worsens the equinus and the supination, and the lateral column of the foot (calcaneus and cuboid) grows more and becomes longer than the medial column (talus, navicular, and cuneiform bones), eventually making the foot very stiff and the deformity no longer reducible [8]. In cases of neglected clubfoot, unsuccessful previous surgery and recurrent deformity, it could be challenging to obtain the correction with manipulation and casting. Therefore, extensive surgery is often required to obtain adequate correction in these cases [9–13]. Different surgical techniques have been advocated for the treatment of unreducible congenital clubfoot, including soft-tissue release, tenotomy and tendon elongation and transfer, joint release, osteotomy and joint fusion; however, the choice of correct surgical procedure for each case is still a cause of concern [14–16]. Moreover, the optimal timing for surgery still remains controversial, although some authors suggest surgery within the first year of life, just before the child starts to walk [17].

We reviewed a series of patients with clubfoot who had undergone nonsurgical and/or surgical treatment. According to the time of diagnosis and the reducibility of the deformity, from reducible deformity observed at birth to unreducible deformity observed after walking age, serial casting and minor or extensive surgical procedures were performed. The aim of this study is to present the treatment of a series of 88 clubfeet observed in newborns, toddlers and ambulating children, and to report the results, early complications and relapse at mid-term follow-up.

## Materials and methods

Eighty-eight clubfeet in 58 patients were observed (bilateral deformity in 30 children). Fifty-two clubfeet (34 patients) were diagnosed at birth, 12 (8 patients) were observed later in non-ambulating children aged between 4 and 12 months, and 24 (16 patients) were observed in ambulating children aged between 1 and 3 years. Clubfeet associated with neurologic or syndromic disorders were not considered in this study. The study was approved by the Ethics Committee and the parents of all the patients provided informed consent to the treatment.

Six non-ambulating children (10 feet) who came to our observation later (between 4 and 12 months) were previously treated at other hospitals with serial casting, whereas two children with unilateral deformity were not previously treated before our observation. Of the 24 clubfeet that came to our observation after walking age, 10 were previously treated with serial casting and 8 with serial casting and posteromedial release at other hospitals, while 6 feet were not previously treated at all.

Clinical assessment of the deformity focused particularly on reducibility of the talo-navicular joint and severity of the equinus. In ambulating children, care was taken to assess the stiffness of the ankle, subtalar and midtarsal joint, and muscular function, strength and atrophy was also evaluated. In these children, pain, skin problems, gait and shoe wearing impairment were also noted.

Each foot was classified using the Pirani score [18]. Radiographic assessment was not performed in newborns and toddlers at the time of diagnosis, whereas it was performed in all feet in ambulating children in order to assess skeletal maturity and to plan surgery.

In all newborns the treatment consisted of manipulation and serial casting according to the Ponseti technique [19]. At the end of casting, residual equinus was corrected by percutaneous Achilles tendon section in all cases but one. During treatment, radiographs in forced ankle dorsiflexion were performed in a 3-month-old child in order to better investigate reducibility of the equinus and confirm that the foot did not require Achilles tenotomy.

Furthermore, in non-ambulating children between 4 and 12 months of age who were observed later, Ponseti serial casting was performed according to recent literature [20]. In 7 feet, the deformity was corrected only by casting and percutaneous Achilles tendon section. However, in 5 feet, Ponseti protocol alone was not sufficient and surgery was performed in order to correct residual deformity after casting around the first year of life: open Achilles tendon lengthening combined with posterior ankle and subtalar joint release was performed in 2 cases in order to correct isolated residual equinus, whereas posteromedial release, performed through a single medial approach according to the technique described by Codivilla [21] was performed in 3 cases to correct residual supination (equinovarus).

Surgical treatment was performed in all ambulating children. In one case that presented isolated relapse of varus after a previous posteromedial release, a selective medial release, including tibialis posterior tendon elongation, talonavicular joint reduction, and abductor hallucis tenotomy was performed combined with cuboid subtraction osteotomy. In 6 feet that presented with partially reducible equinus-varus-supination, with complete reducibility of varus, a posteromedial release was performed. In 17 feet that presented with completely unreducible deformity due to excessive joint stiffness and excessive elongation of the lateral column of the foot, posteromedial release combined with lateral release of the subtalar joint and cuboid subtraction osteotomy fixed with a percutaneous Kirschner wire [22] was performed (Table 1).

**Table 1:** Population data, clinical and radiographic assessment and treatment

Age of observation	No. of feet	Previous treatment			Pirani score			Radiographic assessment	Treatment					
		None	Serial casting	Serial casting + posteromedial release	0–1	1.5–2.5	≥3		Serial casting (Ponseti)	Percutaneous Achilles tenotomy	Open Achilles lenghtening + posterior ankle and subtalar joint release	Selective medial release + cuboid subtractive osteotomy	posteromedial release	posteromedial and lateral release + cuboid subtractive osteotomy
Newborn (0–4 months)	52	52			–	10	42	1 (forced ankle dorsiflexion X-rays at 3 months)	52	51				
Non-ambulating child (4–12 months)	12	2	10		–	2	10	–	12	7	2		3	
Ambulating child (>12 months)	24	6	10	8	–	3	21	24				1	6	17

Clinical evaluation was summarized with the Pirani score. Radiographic assessment was not performed in non-ambulating children except in one case. During treatment with serial casting, radiographs were performed in a 3-month-old child, forcing the ankle in dorsiflexion in order to better evaluate the reducibility of the equinus. In this case, radiographs showed reduction of the equinus and thus there was no need for Achilles tenotomy. All newborns and non-ambulating children were treated with serial casting before performing surgery. All ambulating children underwent surgical treatment

In children who underwent Ponseti serial casting after Achilles tenotomy, a foot-abduction brace was applied full-time for the first 4–6 months, and then only during sleep (continued until 4 years of age) and symmetrical straight last shoes were prescribed when the child started to walk. Furthermore, children who underwent open Achilles tendon elongation followed the same protocol. Children who underwent medial release and posteromedial release (with or without cuboid osteotomy) after surgery wore an above-knee cast for 10 weeks (which was changed after 5 weeks) when the percutaneous Kirschner wire was also removed, followed by application of a foot-abduction brace during the night and symmetrical straight last shoes during walking.

At the last available follow-up each foot was evaluated in terms of plantigrade position, joint stiffness, muscular function, pain, shoewearing and walking. We experienced difficulty in finding a suitable scoring system to assess the results in this case series; however, we decided to use the Pirani score to summarize the results. In cases of plantigrade feet, results were considered as excellent for scores between 0 and 1, good between 1.5 and 2.5, and poor if ≥3; all cases presenting residual equinus were considered poor. The need for further or revision surgery was also noted.

## Results

The average follow-up time was 5 years (range 1–6 years). In newborns who underwent Ponseti serial casting, 4–8 casts (average 6 casts) were necessary to obtain the correction. In non-ambulating children, 6–12 casts (average 8 casts) were made until correction was obtained or surgical treatment was considered necessary to obtain complete correction.

No major complications were reported in children during treatment with serial casting. Sometimes, after cast removal, skin redness or slight swelling of the foot was observed and application of the following cast was delayed by 24–48 h. Neurovascular injury or profuse bleeding after percutaneous Achilles tenotomy were not observed. No infection or wound problems were reported including children who had undergone more extensive surgery. Newborns and toddlers learned to walk between 10 and 19 months and at last follow-up, every child was able to wear shoes and walk without limping, even in cases of residual deformity.

Two ambulating children who had undergone posteromedial release combined with cuboid osteotomy experienced a delay of wound healing; one of these required secondary plastic surgery to restore adequate skin coverage. At last follow-up, two ambulating children who had undergone surgical treatment and presented with residual deformity, reported mild pain during prolonged walking.

**Fig. 1:**
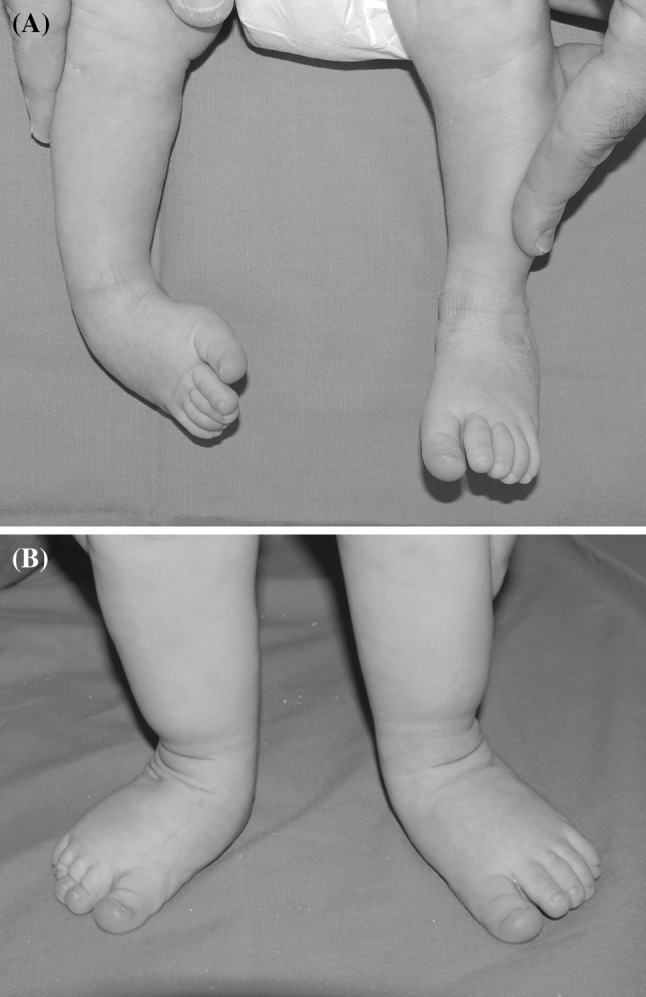
Clinical aspect of monolateral right clubfoot at birth (**a**) and at 18 months after treatment with serial casting and percutaneous Achilles tendon section (**b**)

**Fig. 2:**
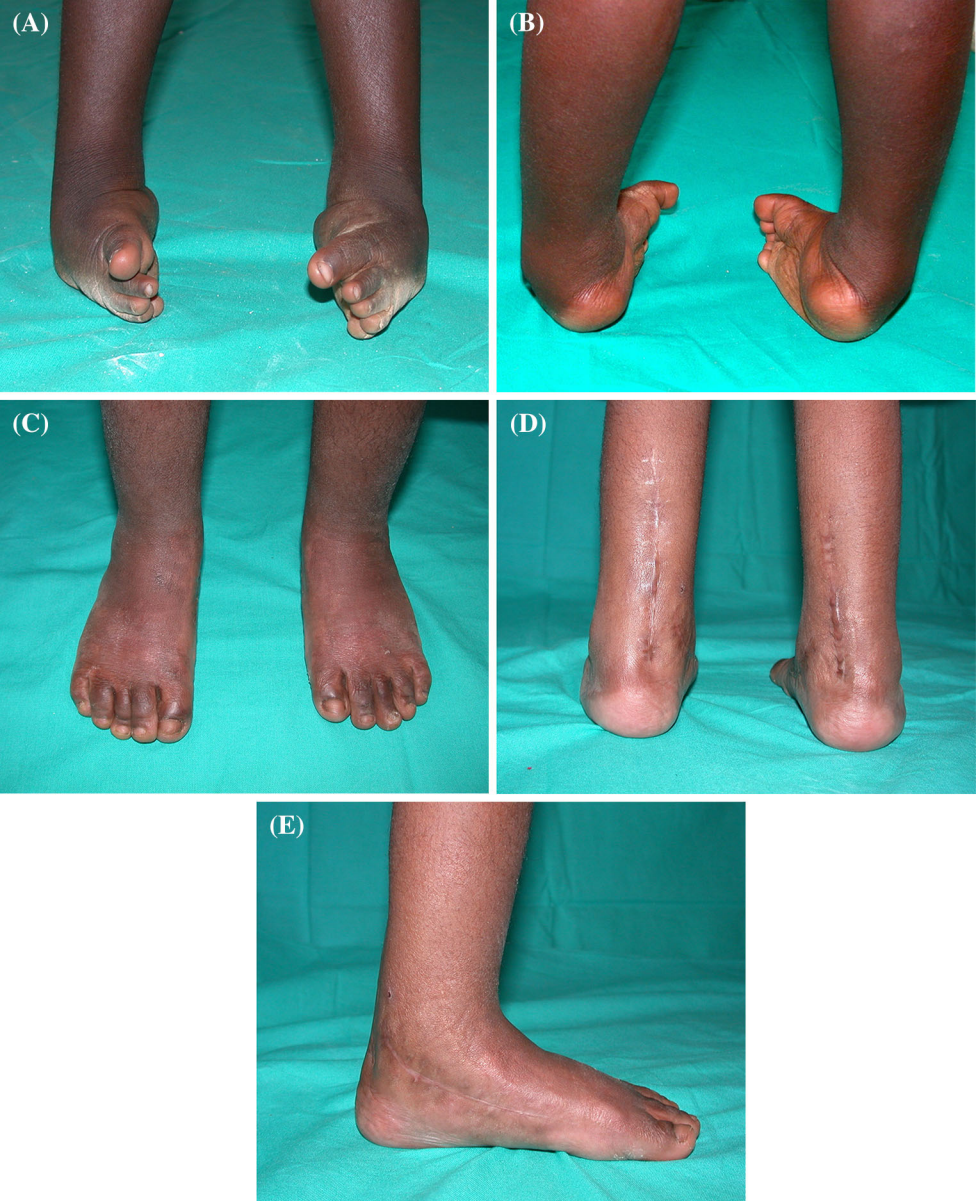
Clinical aspect of bilateral neglected clubfoot in a 3-year-old child presenting with severe rigid deformity (**a**, **b**). Posteromedial release combined with cuboid osteotomy allowed an excellent correction with plantigrade foot (**c**, **d**, **e**)

According to the Pirani score, the results in terms of plantigrade position and residual deformity in newborns treated with Ponseti serial casting were excellent in 42 feet (Fig. 1), good in 6, and poor in 4. In non-ambulating children observed and treated within 4 and 12 months of age, the results were excellent in 9 feet, and good in 3. In ambulating children, the results were excellent in 5 feet (Fig. 2), good in 16, and poor in 3 (Table 2).

**Table 2:** Results

Age of observation (months)	No. of feet	Pirani score		
		Excellent 0–1	Good 1.5–2.5	Poor ≥3
Newborn (0–4)	52	42 (80.7 %)	6 (11.5 %)	4 (7.6 %)
Non-ambulating child (4–12)	12	9 (75 %)	3 (25 %)	–
Ambulating child (>12)	24	5 (20.8 %)	16 (66.6 %)	3 (12.5 %)

According to the Pirani score, the results were excellent for scores between 0 and 1, good between 1.5 and 2.5, and poor for ≥3. All cases presenting with residual equinus were considered poor

In cases that were treated with only the Ponseti method and presented poor results (4 feet), further treatment was performed because of partial relapse observed after the children started walking. In two feet (child age 18 months) with isolated relapse of varus, serial casting was attempted and correction was obtained after 4 casts which were changed every 3 weeks without need of further surgery. In the other two feet (children age 18 and 20 months, respectively) presenting with isolated relapse of equinus, open Achilles tendon lengthening combined with posterior ankle and subtalar joint release was performed. Further surgery was also necessary in cases with poor results (3 feet) in ambulating children surgically treated with posteromedial release. In these cases, relapse of equinovarus was treated with revision of posteromedial release combined with cuboid subtraction osteotomy. No cases of overcorrection were observed in this series.

## Discussion

Currently, most authors suggest conservative treatment for clubfoot, restricting the indication for surgery in cases of recurrent or resistant deformity [23–28]. In newborns, conservative treatment with the Ponseti method is effective in most cases with a high rate of good results [29]. Besides manipulation and casting, minimally invasive surgery consisting of percutaneous Achilles tenotomy, as reported in the literature, very often completes the Ponseti method; in our series all cases but one were treated with this technique. In cases of severe or recurrent equinus, as well as in older children, a greater Achilles retraction could be suspected together with ankle and subtalar joint stiffness. In these cases we assumed that percutaneous Achilles tenotomy alone could not obtain satisfactory correction; hence, a little more extensive surgery was performed in some cases. Some authors also suggest performing percutaneous Achilles tendon section in ambulating children and in cases of relapse of equinus while others advise open lengthening or use of percutaneous incisions as described by Hoke [30–32]. We preferred to perform open Achilles tendon lengthening in older children or in cases of relapse of the equinus in order to also appreciate the possible tightness of the ankle and subtalar joints and then perform a posterior release of these joints along with tendon lengthening.

In our series some children were observed and treated later, after 4 months of age and before they started walking. In these patients, serial casting was performed according to Ponseti as well as in newborns. However, these cases revealed stiffer deformities which were more challenging to treat. The number of casts necessary to obtain correction was higher than in newborns (6–12 casts versus 4–8 casts) and in some cases (5 of 12) complete correction was not achieved. Therefore, we decided to perform surgery before the children start walking. As reported, isolated equinus was treated with open Achilles tendon lengthening and posterior ankle and subtalar release, while in cases of residual supination (equinovarus) we decided to perform a posteromedial release according to the technique described by Codivilla . This technique allows the release of retracted tissues on the medial aspect of the foot, restores congruency of the talonavicular joint (and naviculocuneiform and cuneometatarsal joints if necessary), along with correction of the equinus by Achilles tendon lengthening and posterior release of the ankle and subtalar joint.

As suggested by some authors, conservative treatment with manipulation and casting should be attempted not only in older children but also in cases of neglected or relapsed clubfoot, even if this treatment does not always ensure a complete correction and prevent the need for surgery [33–37]. In our series, serial casting in the treatment of partial relapse after Ponseti treatment proved to be an encouraging option, even though our experience with this kind of approach is still very limited to date.

We always performed surgery in ambulating children. Posteromedial release resulted adequate to obtain complete correction of the deformity, when complete correction of the forefoot varus was achieved using medial release. Conversely, in severe neglected clubfeet, posteromedial release alone was not sufficient to completely correct the deformity. Excessive growth of the lateral column of the foot compared with the medial column, that is shorter as a consequence of tissues retraction and joint dislocation, produces a more severe equinovarus deformity. Furthermore, in older children, ambulation enhances stiffness of the deformity. In these cases, posteromedial release alone was not sufficient to obtain a complete correction. Lateral release and cuboid subtraction osteotomy combined with posteromedial release rebalanced the length of medial and lateral columns of the foot, thus helping complete reduction of the talonavicular joint and allowing complete correction of forefoot varus. Posteromedial release combined with cuboid subtraction osteotomy also proved to be a viable option in cases of relapse after extensive surgical treatment. In cases of less severe deformity, as well as in those feet still presenting residual or relapsed isolated varus, following the same principles of the described techniques, we believe that cuboid subtraction osteotomy can be combined with selective medial release in order to obtain the correction.

Many complications have been reported after extensive surgical treatment of clubfoot, mainly incomplete corrections or overcorrections, skin problems and neurovascular injuries. Moreover, some authors have reported a loss of correction over time and residual deformity after skeletal maturity, stiffness and/or early degenerative changes involving the ankle, subtalar and midtarsal joints, pain and muscle weakness; therefore, they do not recommend surgery as a primary treatment for clubfoot [38]. Based on our experience, surgical treatment should be considered in walking children because of the greater stiffness of the foot, mainly in cases of obvious deformity in which the lateral column of the foot is much longer than the medial as a consequence of an unbalanced growth. Surgical treatment allowed us to obtain a satisfactory rate of good results with few complications and low rate of deformity relapse at mid-term follow-up.

It is still difficult to determine appropriate timing and technique for the surgical treatment of congenital clubfoot, and the approach for both primary and revision surgery has been often defined as ‘a la carte’ [39]. The age of the patient at the time of diagnosis, walking age, stiffness of the deformity and previous treatment are all major concerns in the choice of appropriate treatment in order to determine the need for surgery rather than nonsurgical treatment, particularly within the first year of age. There are some remarkable limitations to this study, mostly concerning the non-homogeneous series of patients, the different grades of deformity, untreated and previously treated patients, and the relatively short follow-up. In our experience, newborns and toddlers can be successfully treated nonsurgically with serial casting, even though this treatment seems to become more challenging as the child ages or if it is not started just after birth. Conversely, we advise surgical treatment after walking age. Nevertheless early reports about re-casting with the Ponseti technique seem encouraging, extending the opportunity for nonsurgical or less-invasive surgical procedures (i.e., Achilles tendon lengthening) in older children and in cases of relapse and more severe deformities.
